# Transcription of the Human 5-Hydroxytryptamine Receptor 2B (HTR2B) Gene Is under the Regulatory Influence of the Transcription Factors NFI and RUNX1 in Human Uveal Melanoma

**DOI:** 10.3390/ijms19103272

**Published:** 2018-10-21

**Authors:** Manel Benhassine, Sylvain L. Guérin

**Affiliations:** 1 Centre Universitaire d’Ophtalmologie-Recherche (CUO-Recherche), Axe médecine régénératrice, Hôpital du Saint-Sacrement, Centre de Recherche FRQS du CHU de Québec, Université Laval, Québec, QC G1S4L8, Canada; manal.benhassine.1@ulaval.ca; 2 Département d’ophtalmologie, Faculté de Médecine, Université Laval, Québec, QC G1V0A6, Canada

**Keywords:** *HTR2B*, gene promoter, nuclear factor I (NFI), Runt-related transcription factor I (RUNX1), uveal melanoma

## Abstract

Because it accounts for 70% of all eye cancers, uveal melanoma (UM) is therefore the most common primary ocular malignancy. In this study, we investigated the molecular mechanisms leading to the aberrant expression of the gene encoding the serotonin receptor 2B (HTR2B), one of the most discriminating among the candidates from the class II gene signature, in metastatic and non-metastatic UM cell lines. Transfection analyses revealed that the upstream regulatory region of the *HTR2B* gene contains a combination of alternative positive and negative regulatory elements functional in *HTR2B*^−^ but not in *HTR23B*^+^ UM cells. We demonstrated that both the transcription factors nuclear factor I (NFI) and Runt-related transcription factor I (RUNX1) interact with regulatory elements from the *HTR2B* gene to either activate (NFI) or repress (RUNX1) *HTR2B* expression in UM cells. The results of this study will help understand better the molecular mechanisms accounting for the abnormal expression of the HTR2B gene in uveal melanoma.

## 1. Introduction

Uveal melanoma (UM), with an incidence of 4 to 6 affected individuals per million in the United States [1], accounts for 70% of all eye cancers, which makes it the most observed ocular malignancy among the adult population. Although the primary tumor can be efficiently treated, the 15-year mortality due to dissemination of the cancer cells to the liver has reached approximately 50% [2]. Cumulative microarray analyses identified 12 genes, designated as the UM gene expression signature, that can distinguish between UM primary tumors that are at low (Fragile X mental retardation syndrome-related protein 1 (*FXR1*), leukotriene A4 hydrolase (*LTA4H*), DNA-binding protein inhibitor ID-2 (*ID2*), Roundabout homolog 1 (*ROBO1*), Mitochondrial tumor suppressor 1 (*MTUS1*), LIM and cysteine rich domains 1 (*LMCD1*), stabilin 1 (*STAB1*) and eukaryotic translation initiation factor 1B (*EIF1B*); designated as Class I) or high (*HTR2B*, Cadherin 1 (*CDH1*), RAB31, member RAS oncogene family (*RAB31*) and Extracellular Matrix Protein 1 (*ECM1*), designated as Class 2) risk of progressing towards the liver metastatic disease [3]. The eight-year survival probability has been established to be 95% in class 1 patients against only 31% in class 2 patients [3]. The human gene encoding the 5-Hydroxytryptamine receptor 2B (*HTR2B*), also known as the serotonin receptor 2B, turn out to be the most discriminating among the class II genes in order to identify UM patients at high risk of evolving toward the liver metastatic disease [4,5].

The serotonin receptor HTR2B belongs to a larger family of proteins that comprises seven sub-families (HTR1 to HTR7) [6]. When it binds its ligand serotonin (5-HT), HTR2B activates the G proteins GNAQ, GNA11 and GNA13, and participates in the development and cell proliferation and survival through the activation of a few signal transduction pathways such as the phospholipase C (PLC), Janus kinase/Signal Transducer and Activator of Transcription proteins (JAK/STAT), Receptor Tyrosin Kinase (RTK)/Phosphatidylinositol-4,5-bisphos- phate-3-kinase (PI3K)/Extracellular signal-Regulated Kinase (ERK)/mammalian target of rapamycin (mTOR) and RAF/Mitogen activated protein Kinase Kinase (MEK)/ERK pathways [6,7,8,9,10]. By preventing differentiation of the neural crest cells, HTR2B also plays a role in embryonic morphogenesis [11]. A few studies ascribed mitogenic and angiogenic properties to serotonin [12,13,14,15]. Curiously, the HTR2B receptor has been described as an oncogene in certain types of cancers (hepatocellular and prostate cancers) [15,16,17], but also as a tumor suppressor in others (such as ovarian cancers) [18]. Interestingly, excess serotonin signaling that results from the overexpression of the HTR2B receptor was found to cause the formation of irregularly shaped eyes that are inappropriately positioned and oriented in Xenopus embryos [19].

Although the signal transduction cascades activated by the binding of serotonin to HTR2B has been investigated for years, no study has ever reported the characterization of the regulatory sequences that are critical to ensure proper transcription of the *HTR2B* gene. Meanwhile, no survey has ever explored the mechanistic of the deregulated *HTR2B* gene expression at the transcription level in the class 2 tumors at risk of progressing towards the metastatic liver disease. In order to better understand the molecular mechanisms involved in the deregulated expression of HTR2B, we characterized both the transcription factors (TFs) and the regulatory elements to which they bind, that are important for its expression in human UM cells that express this gene to different levels.

## 2. Results

### 2.1. HTR2B Expression in Human Uveal Melanoma Cell Lines

**Figure 1:**
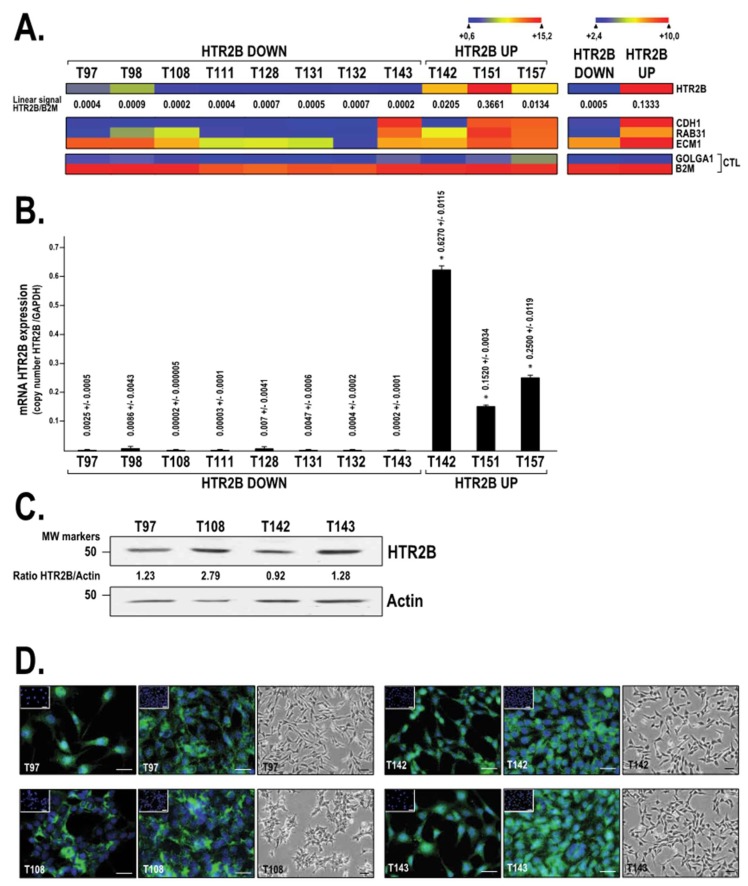
Expression of HTR2B in UM cell lines (**A**) Heatmap representation of the transcriptional profiles of the class 2 genes from the uveal melanoma gene signature (*HTR2B*, *CDH1*, *RAB31* and *ECM1*) expressed by UM cell lines. HTR2B Down: UM cell lines that express either no or only very low levels of *HTR2B*; HTR2B Up: UM cell lines that express moderate to high levels of *HTR2B*. Data are also shown for β2-microglobulin (*B2M*) and golgin subfamily A member 1 (GOLGA1), two housekeeping genes whose expression are very high and low, respectively, in all cell types. A dark blue color corresponds to a very low level of HTR2B gene expression, whereas high levels appear in yellow/red. (**B**) qPCR analysis of *HTR2B* expression in cells used on Panel A. Data are presented as the ratio of *HTR2B* mRNA copy number over that of the *GAPDH*. (**C**) Western blot analysis of *HTR2B* expression in T97, T108, T142 and T143 cells. Actin expression was monitored as a normalization control. (**D**) Immunofluorescence analysis of HTR2B expression (in green) in the UM cell lines T97, T108, T142 and T143 grown to sub- (**left panel**) or mid-confluence (**middle panel**). Phase contrast micrographs are also provided for each cell line (**right panel**). Insets: no addition of the primary antibody. Nuclei appear in blue. Scale bar: 20 µM.

Gene profiling on microarrays was first exploited in order to monitor the expression of the four candidates (*HTR2B*, *CDH1*, *RAB31* and *ECM1*) that belong to the UM class II gene signature in a variety of UM cell lines cultured at low passage (P1 to P16). As shown in Figure 1A, UM cell lines that express high levels of *HTR2B* also often express elevated levels of the *CDH1*, *RAB31* and *ECM1* genes (ratio of signal normalized to the internal control β2-microglobulin (RNS) ranging from 0.0002 to 0.3661; Figure 1A, left). The highest normalized level of *HTR2B* expression was observed in the UM cell lines T142 (RNS: 0.0205), T151 (RNS: 0.3661) and T157 (RNS: 0.0134), whereas the lowest levels were observed in T97, T98, T108, T111, T128, T131, T132, and T143 cells (with RNS of 0.0004, 0.0009, 0.0002, 0.0004, 0.0007, 0.0005, 0.0007 and 0.0002, respectively). When analyzed as replicates (Figure 1A, right), low *HTR2B*-expressing UM cell lines had an RNS of 0.0005 ± 0.0002, whereas it was of 0.1333 ± 0.2016 for high *HTR2B*-expressing UM cell lines (which corresponds to a 267-fold difference between low and high *HTR2B*-expressing UM cells). The variations in *HTR2B* expression observed by microarrays between UM cell lines were also validated by quantitative PCR (qPCR) (Figure 1B). However, despite the fact that T97, T108 and T143 cells were found to express no or very low levels of the *HTR2B* transcript (as revealed by both gene profiling and qPCR analyses), a significant amount of HTR2B protein was observed by Western blot (and further validated by indirect immunofluorescence) in these UM cell lines (as well as in T142; Figure 1C,D). Interestingly, normalization of the *HTR2B* signal to that of the actin internal control provided evidence that T108 cells, which express a very low, barely detectable *HTR2B* mRNA level, also have the highest normalized level of HTR2B protein (ratio of 2.79; Figure 1C). This result is also consistent with the higher, more uniform and less diffused signal obtained in immunofluorescence analysis for T108 cells (Figure 1D). On the other hand, T142 cells, which express the highest level of *HTR2B* at the mRNA level, had the lowest normalized ratio of HTR2B protein (0.92; Figure 1C), therefore suggesting that a reverse relationship, through the use of a negative feedback loop, may exist between the expression of HTR2B at the mRNA and protein levels.

**Figure 2:**
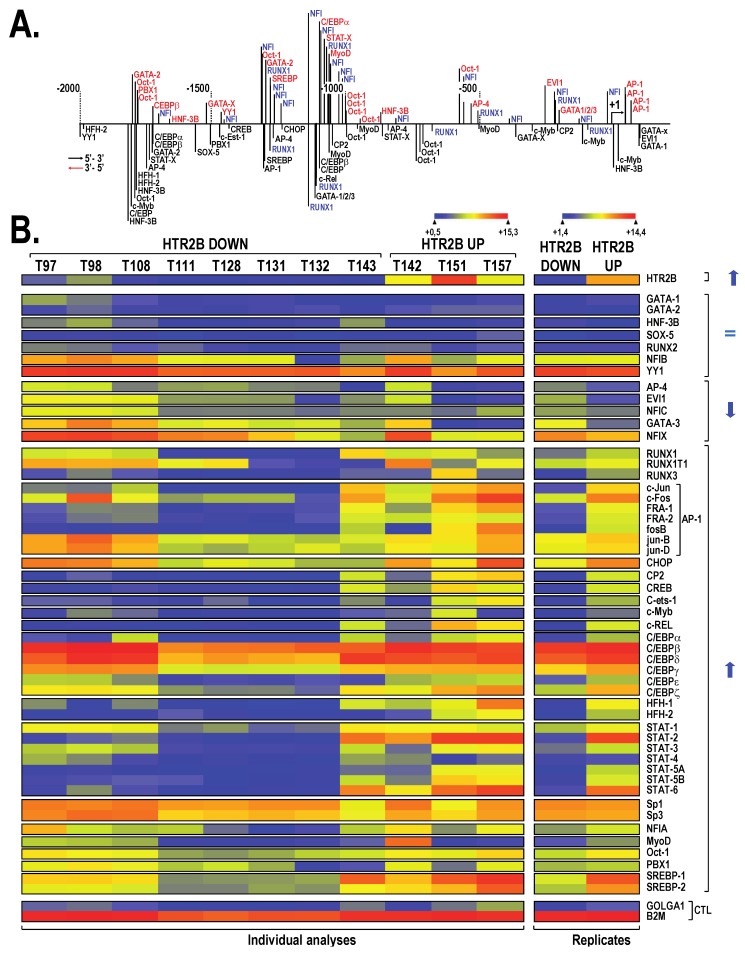
Putative transcription factor binding sites along the *HTR2B* promoter and 5′-flanking sequence. (**A**) Schematic representation of the human *HTR2B* promoter and 5′-flanking sequence along which presumed transcription factor binding sites are indicated. TF target sites identified on the DNA top strand are indicated in red and blue whereas those indicated in black are located on the bottom strand. (**B**) Heatmap representation of the transcriptional profiles of all the TFs expressed by UM cell lines for which a putative target site was identified in panel (**A**) and which express either low (HTR2B Down) or high levels of *HTR2B* (HTR2B Up). They are also expressed as *HTR2B* down and up replicates (**right column**). Microarray data for *B2M* and *GOLGA1* are also shown.

We then subjected a segment from the *HTR2B* gene extending up to approximately 2 Kbp upstream from the HTR2B mRNA start site to a search with the TFSEARCH program, a tool that can identify putative DNA target sequences for most nuclear-located TFs. Target sites for 25 different TFs (or families of TFs) that can potentially bind the *HTR2B* promoter were identified using this program (Figure 2A). Interestingly, a particularly high number of putative target sites were identified for the TFs runt related transcription factor 1 (RUNX1) (9 sites) and Nuclear Factor I (NFI) (17 sites), two TF families that have been reported to function either as activators or repressors of gene expression [20,21,22,23,24]. We next examined the pattern of expression for each of these TFs in the different UM cell lines that also express the *HTR2B* gene to different levels by searching the microarray data files used for generating the data appearing in Figure 1A. As shown in Figure 2B, some of these TFs, such as GATA protein 1 and 2 (GATA-1 and GATA-2), forkhead box A2 (HNF-3B), SRY-box 5 (SOX-5), runt related transcription factor 2 (RUNX2) and MYB proto-oncogene transcription factor (c-Myb) are either not expressed or only barely detectable in all UM cell lines and thus are not worth paying them too much attention. The low *HTR2B* expressing cells (T97, T98, T108, T111, T128, T131, T132 and T143) distinguish themselves from those that moderately or highly express that gene (T142, T151 and T157) by their lower level of expression of the TFs RUNX1, nuclear factor IA (NFIA), some of the CCAAT/enhancer binding protein (C/EBP) and signal transducer and activator of transcription (STAT) family members, and all the activator protein-1 (AP-1) constituting subunits (most particularly Jun proto-oncogene (c-Jun), Fos proto-oncogen (c-Fos), P-loop containing nucleoside triphosphate hydrolases superfamily proteins 1 and 2 (FRA-1 and FRA-2) and FosB proto-oncogen (FosB) (Figure 2B, right column (replicates)), therefore suggesting that these TFs might contribute to the variations observed in the expression of the *HTR2B* gene between UM cell lines.

### 2.2. HTR2B Gene Transcription Is Modulated by Both Positive and Negative Regulatory Elements

**Figure 3:**
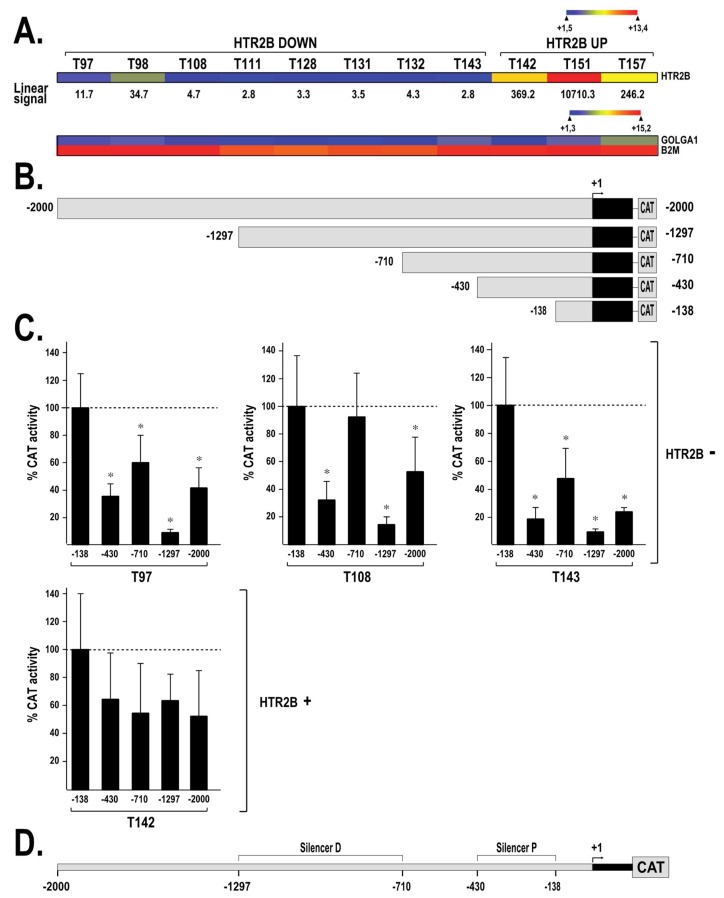
Transfection of the *HTR2B* gene promoter in UM cell lines. (**A**) Heatmap representation of *HTR2B* expression in UM cell lines. The values of the *HTR2B* linear signals are also provided. (**B**) Representation of the HTR2B/CAT recombinant plasmids used for transfection analyses. Numbers indicate position relative to the *HTR2B* theoretical mRNA start site (indicated by a curved arrow). (**C**) CAT activities measured following transfection of the *HTR2B* constructs shown in panel B in the UM cell lines that express either low (*HTR2B*^−^; T97, T108 and T143) or high (*HTR2B*^+^; T142) levels of *HTR2B*. CAT activity is expressed relative to the level directed by the HTR2B/−138 construct. *: Values considered to be statistically significant from those obtained with the HTR2B/−138 construct (*P* value < 0.001). (**D**) Schematic representation of both the proximal (Silencer P) and distal (Silencer D) silencers along the *HTR2B* promoter and 5′-flanking region based on the transfection results from panel (**B**).

We then transfected plasmids bearing different segments from the *HTR2B* gene promoter and 5′-flanking sequences fused to the chloramphenicol acetyl transferase (CAT) reporter gene (Figure 3B) into UM cells in order to help us determine the position of the regulatory elements that contribute to basal expression of the *HTR2B* gene. Transfection of the plasmid HTR2B/-138 that contains the *HTR2B* promoter sequence from 3′ position +96 to 5′ position −138 relative to the transcriptional start site into UM cell lines that express either very low (T97, T108 and T143) or intermediate (T142) levels of *HTR2B*, respectively (Figure 3A), yielded easily detectable CAT activities in all UM cell lines (Figure 3C). However, extending the *HTR2B* promoter to position −430 (in plasmid HTR2B/−430) caused a significant 2.7- to 5.3-fold repression in the HTR2B-negative UM cell lines T97, T108 and T143 but not in the HTR2B-positive T142 cells (Figure 3C). Extending the *HTR2B* promoter by 280 bp to position −710 (in HTR2B/−710)) partly relieved this repression in T97, T108 and T143 cells, but had no influence in T142 cells. Extending further the *HTR2B* promoter to position −1297 resulted in a strong repression (7- to 12-fold repression) of the CAT activity relative to the level directed by the HTR2B/−138 construct in T97, T108 and T143, but not in T142 cells. Again, this repression was partly released upon transfection of the plasmid HTR2B/−2000 in T97, T108 and T143 cells. Collectively, these results indicate clearly that transcription of the *HTR2B* gene is under the control of two negative silencer elements: a strong one (designated as the distal silencer (Silencer D)) located between positions −710/−1297, and a weaker one (designated as the proximal silencer (Silencer P)) present between positions −138/−430 (Figure 3D). They also suggest that regulatory elements required to ensure basal expression of *HTR2B* are present between positions +96 and −138.

### 2.3. Members from the NFI Family Bind to the HTR2B Promoter and Positively Regulate Its Transcriptional Activity in Uveal Melanoma

**Figure 4:**
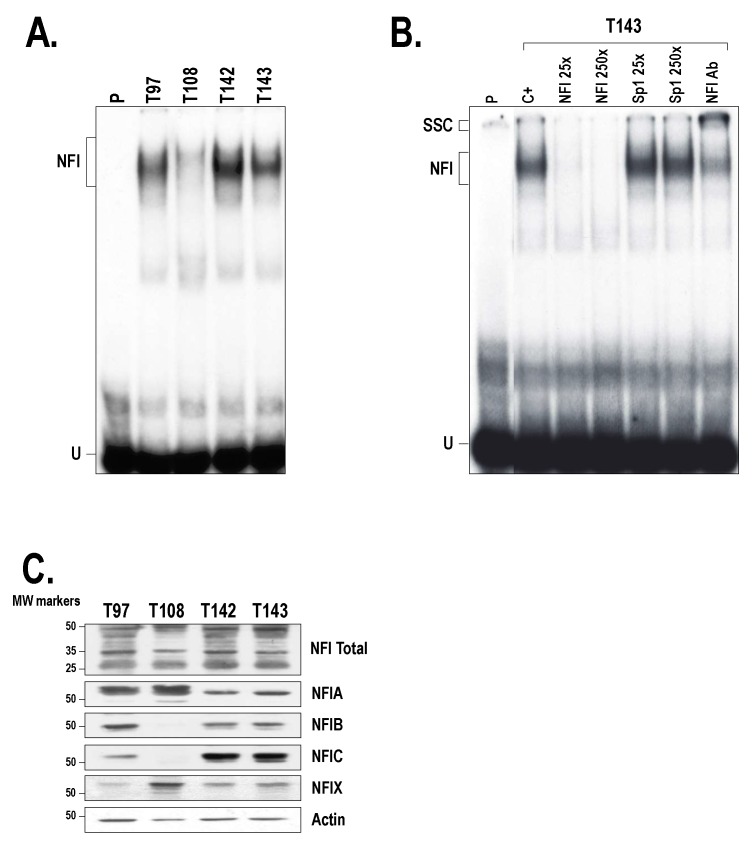
Expression of NFI in the UM cell lines. (**A**) Nuclear proteins (5 µg) obtained from the UM cell lines T97, T108, T142 and T143 were incubated with a 5′ end-labeled probe bearing the high affinity binding site for NFI. Formation of DNA-protein complexes was then monitored by EMSA. The position of the NFI complex is indicated along with that of the free probe (U). P: labeled probe without added proteins. (**B**) Nuclear proteins from T143 cells were incubated with the NFI probe either alone (C+) or in the presence of a 25- or 250-fold molar excess of unlabeled competitor oligonucleotides bearing the target site for either NFI or Sp1. When indicated (last track), a polyclonal antibody that can recognize all NFI isoforms (NFI Ab) was added along with T143 nuclear proteins. SSC: supershifted complex formed upon addition of the NFI antibody. (**C**) Western blot analysis of the NFI isoforms in the UM cell lines T97, T108, T142 and T143. UM cell nuclear extracts were also blotted using an antibody that can recognizes all four NFI isoforms (NFI Total). Actin expression was monitored as a normalization control.

To assess whether UM cell lines express NFI in vitro, nuclear extracts were prepared from T97, T108, T142 and T143 cells and used in electrophoretic mobility shift assay (EMSA). Incubation of nuclear proteins from all UM cell lines with a labeled probe bearing the high affinity NFI target site revealed the formation of a diffuse complex typical of NFI binding to DNA (Figure 4A). Although equal amounts of nuclear proteins (5 µg) were used for the assay, the extract from T108 cells yielded only a weak DNA-protein signal on gel, whereas that from T142 generated the strongest shifted complex. Addition of either a 25- or 250-fold molar excess of the unlabeled NFI oligonucleotide, but not that of a double-stranded oligonucleotide bearing the target site for the unrelated transcription factor Sp1, entirely eliminated formation of the specific NFI complex (Figure 4B). The significant reduction of the NFI complex combined to the formation of a new, slow-migrating supershifted complex (SSC) upon addition of a polyclonal antibody that recognizes all NFI isoforms (Figure 4B; NFI Ab) confirmed its specificity of formation.

To decipher which of the four NFI isoforms are expressed by our UM cell lines, nuclear proteins prepared from T97, T108, T142 and T143 UM cells were Western blotted using antibodies specific to the NFIA, -B, -C and -X isoforms. An antibody that can recognize all NFI isoforms (NFI Total) has also been used as a control. As shown in Figure 4C, T97, T142 and T143 cells express all four NFI isoforms, although to different levels (T97 primarily express both NFIA and NFIB whereas T142 and T143 cells predominantly express the NFIC isoform). On the other hand, T108 only expresses the NFIA and NFIX isoforms. The multiple bands detected for some of the NFI isoforms (particularly noticeable for both NFIA and NFIC) are most likely indicative of post-translational modifications occurring in some UM cell lines (for instance, T97 and T108 for NFIA and T142 and T143 for NFIC).

**Figure 5:**
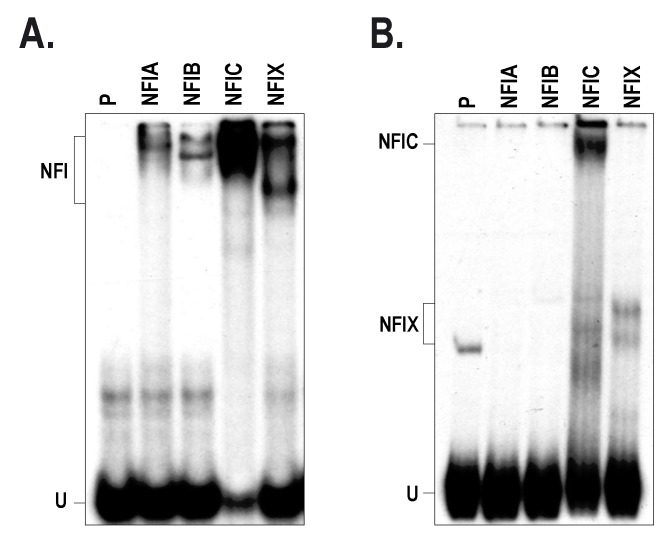
Differential binding of the NFI isoforms to the HTR2B silencer D region. (**A**) The NFI labeled probe used in Figure 4A was incubated with equal amounts of bacterially produced, recombinant NFIA, NFIB, NFIC and NFIX proteins prior to analysis of the DNA-protein complexes by EMSA (8% native polyacrylamide gel run at 40 V for 18 h). U: free probe; P: labeled probe without added proteins. (**B**) Same as in panel A except that a 278 bp DNA fragment bearing most of the *HTR2B* distal silencer from position −1037 to −1315 was used as the probe in the EMSA (6% native polyacrylamide gel run at 80 V for 18 h).

The −710/−1297 distal silencer bears the highest number of putative NFI target sites identified along the *HTR2B* promoter and 5′-flanking sequence (10 out of 17; Figure 2A). We therefore examined the ability of each of the four NFI isoforms to interact with the *HTR2B* distal silencer element by EMSA. To that purpose, we 5′ end-labeled a 278 bp BglII/BstXI restriction fragment bearing a large segment from the *HTR2B* sequence (from position −1037 to −1315; 7 NFI sites) and incubated this labeled probe with bacterially produced, recombinant NFIA, NFIB, NFIC and NFIX proteins prior to analysis of the DNA-protein complexes by EMSA. As shown in Figure 5A, incubation of each of the recombinant NFI isoforms with the labeled probe bearing the prototypical, high affinity NFI binding site used in Figure 4 yielded a typical NFI-protein complex on the EMSA. However, when the NFI labeled probe was replaced by the *HTR2B* 278 bp Silencer D labeled probe, only the NFIC and NFIX isoforms could yield a shifted DNA-protein complex on gel (Figure 5B; the position of the complexes yielded by NFIC and NFIX is different from that obtained in panel A because of the variation in the size of the labeled probe used (26 bp in panel A and 278 bp in panel B), which also required a much-longer time of gel migration in panel B in order to bring the free probe to the bottom of the gel). The NFIC and NFIX isoforms have been shown to be expressed at both the mRNA (Figure 2B) and protein (Figure 4C) level in most of our UM cell lines.

**Figure 6:**
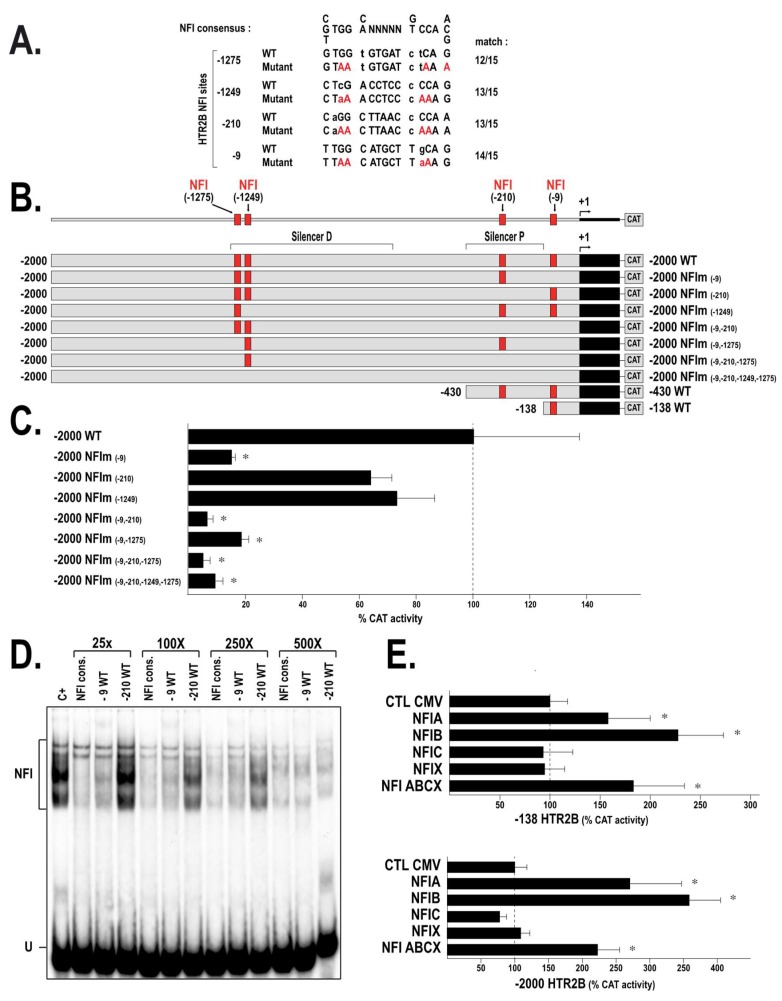
Transfection of the mutated NFI constructs in the UM cell lines. (**A**) DNA sequence of four NFI target sites identified in the *HTR2B* regulatory region at position −9, −210, −1249 and −1275, aligned with the NFI prototypical sequence. The nucleotides preserved between the *HTR2B* NFI sites and the NFI consensus sequence are shown in capital letters along with those selected for site-directed mutagenesis (mutations are shown in red). (**B**) Schematic representation of the HTR2B/−2000, HTR2B/−430 and HTR2B/−138 wild-type (WT) parental plasmids along with their derivatives that have been mutated in one or more of the NFI sites shown in panel A (the NFI sites preserved in each construct are shown in red). (**C**) CAT activities measured following transfection of the *HTR2B* constructs in T108 cells. CAT activity is expressed relative to the level directed by the parental construct HTR2B/−2000. *: Values statistically significant from those obtained with the wild type, parental construct (*P* value < 0.001). (**D**) Nuclear proteins (15 µg) from 293T cells incubated with the NFI probe either alone (C+) or in the presence of a 25- to 500-fold molar excess of unlabeled competitor oligonucleotides bearing the prototypical NFI site (NFI cons.) or the NFI sites identified at position −9 (−9WT) or −210 (−210WT) in the HTR2B gene promoter. Formation of the NFI DNA-protein complexes was then examined by EMSA (**E**) The HTR2B/−2000 and HTR2B/-138 plasmids were co-transfected in T108 cells with plasmids encoding high levels of each of the NFI isoforms (either individually or in combination (NFI-ABCX)) or with the pLenti6V5A empty vector (CtlCMV). CAT activities were measured and expressed relative to the level directed by the parental constructs HTR2B/−2000 and HTR2B/−138. *: Values considered to be statistically significant from those obtained with the corresponding wild type, parental construct (*P* value < 0.001).

In order to evaluate whether binding of NFI proteins to the target sites identified in the *HTR2B* 5′ regulatory sequences function as activators or repressors of gene transcription, we identified those whose sequence is the closest from the prototypical NFI target site [25,26] and used site-directed mutagenesis combined with transfection experiments to evaluate their contribution to the *HTR2B* promoter activity. Four putative target sequences (located at positions −9, −210, −1249 and −1275 relative to the *HTR2B* mRNA start site) with identities to the prototypical NFI sequence ranging from 12 to 14 preserved nucleotides (relative to the 15 residues of the prototypical NFI site) were therefore chosen (Figure 6A). Each of these NFI sites was mutated in the *HTR2B* promoter-bearing plasmids, either individually or in combination, and the derivative constructs transfected in T108 UM cells. As indicated in Figure 6C, mutation of the −9 NFI site in the plasmid −2000/NFIm(−9) yielded a near 7-fold reduction in T108 cells when compared to its parental vector HTR2B/−2000 (considered as 100%). On the other hand, mutating either the −210 (in −2000/NFIm(−210)) or the −1249 (in −2000/NFIm(−1249)) NFI site reduced CAT activity by only 40% and 27% in these cells, respectively. Interestingly, mutation of both the −9 and −210 NFI sites in the plasmid −2000/NFIm(−9, −210) caused a dramatic reduction of the CAT activity to only 6% (17-fold repression) relative to the level driven by the parental plasmid −2000/HTR2B. Mutating both the −1279 and −9 sites in −2000/NFIm(−9, −1249) did not reduce promoter activity beyond the level of the −9 NFI mutant alone (18% vs. 15%, respectively) suggesting that the regulatory impact of the −1275 NFI site is negligible. Similarly, mutating the −1275 NFI site along with both the −9 and −210 sites in plasmid −2000/NFIm(−9,−210,−1275) did not reduce further the CAT activity (5% of the level directed by the parental plasmid −2000/HTR2B) than the level observed with the plasmid −2000/NFIm(−9,−210). Mutation of all four NFI sites in −2000/NFIm(−9,−210,−1249,−1275) reduced CAT activity to very much the same level (9%) as that yielded by the −9/−210 double mutant (6%). These results suggest that most of the positive influence exerted by the TF NFI is mediated by the −9 site, whereas the three remaining NFI sites only exert a marginal influence on the *HTR2B* promoter under the conditions used.

Competitions in EMSA using increasing amounts (25- to 500-fold molar excesses) of unlabeled double-stranded oligonucleotides bearing the DNA sequence of either the −9 or −210 HTR2B NFI site revealed that the *HTR2B* −9 NFI oligonucleotide was as efficient as the prototypical NFI site to compete for the formation of the NFI DNA-protein complex yielded by the nuclear extract prepared from 293T cells (which has been selected as the source of proteins because they express high levels of NFI; Figure 6D). On the other hand, the −210 NFI oligonucleotide was 20-times less effective (as revealed by the ratio of the counts per minute (cpm) from the shifted NFI DNA-protein complex over the total cpm counts) than the −9 oligonucleotide at competing for the formation of the NFI complex. This is consistent with the fact that the −9 NFI site deviates from the consensus NFI site by only one nucleotide, whereas the −210 site is different from the consensus by 2 nucleotides (Figure 6A).

We next transfected the wild-type HTR2B/−138, HTR2B/−430 and HTR2B/−2000 constructs in T108 cells along with expression vectors encoding each of the NFI isoforms. As shown in Figure 6E, the basal HTR2B/−138 plasmid (this plasmid was selected because it is deleted from the −210 NFI site and therefore bears only the −9 NFI site) responded to both NFIA (increase to 168 ± 33%) and NFIB (increase to 227 ± 46%) in T108 cells but not to NFIC and NFIX. Its activity also increased (to 184 ± 52%) when all four NFI isoforms were co-transfected along with HTR2B/−138. Similarly, the HTR2B/−2000 plasmid responded very efficiently to both NFIA (increase to 286 ± 61%) and NFIB (increase to 358 ± 57%) but not to NFIC and NFIX whereas its activity also increased (to 220 ± 35%) when co-transfected along with all four NFI isoforms.

### 2.4. The Transcription Factor RUNX1 Binds to the HTR2B Promoter In Vitro

**Figure 7:**
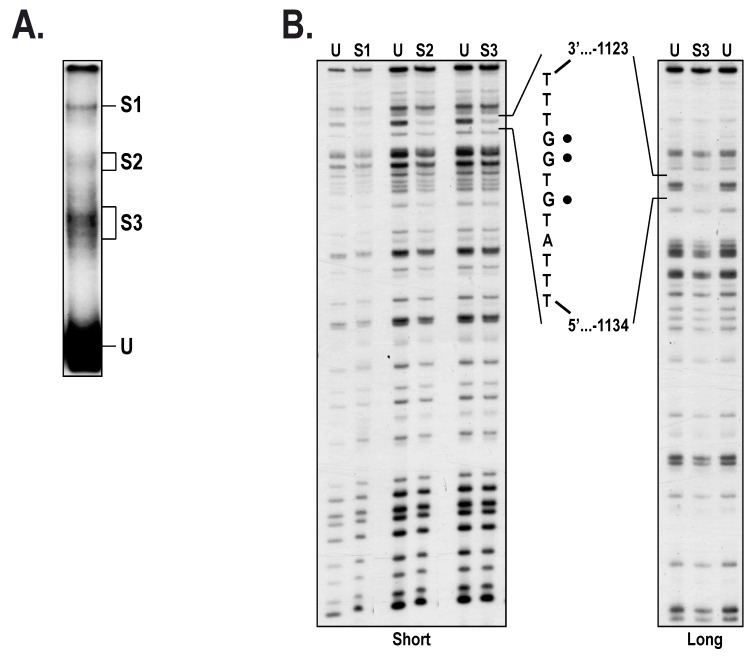
In vitro DMS footprinting of RUNX1 binding to the HTR2B promoter. (**A**) The 278 bp −1037/−1315 BglII/BstXI restriction fragment bearing most of the *HTR2B* distal silencer sequence used in Figure 5B was 5′ end-labeled, methylated with DMS and incubated with 30 µg nuclear proteins from T142 cells before the DNA/protein complexes were separated by EMSA. (**B**) Labeled DNA isolated from the S1, S2 and S3 complexes and the free probe (U) and treated with piperidine before analysis on a sequencing gel. Labeled DNA samples were separated at 1800 V for either 90 (short run) or 180 min (long run). The DNA sequence from the *HTR2B* promoter that includes the protected G residues (identified by black dots) is indicated along with its positioning relative to the *HTR2B* mRNA start site.

**Figure 8:**
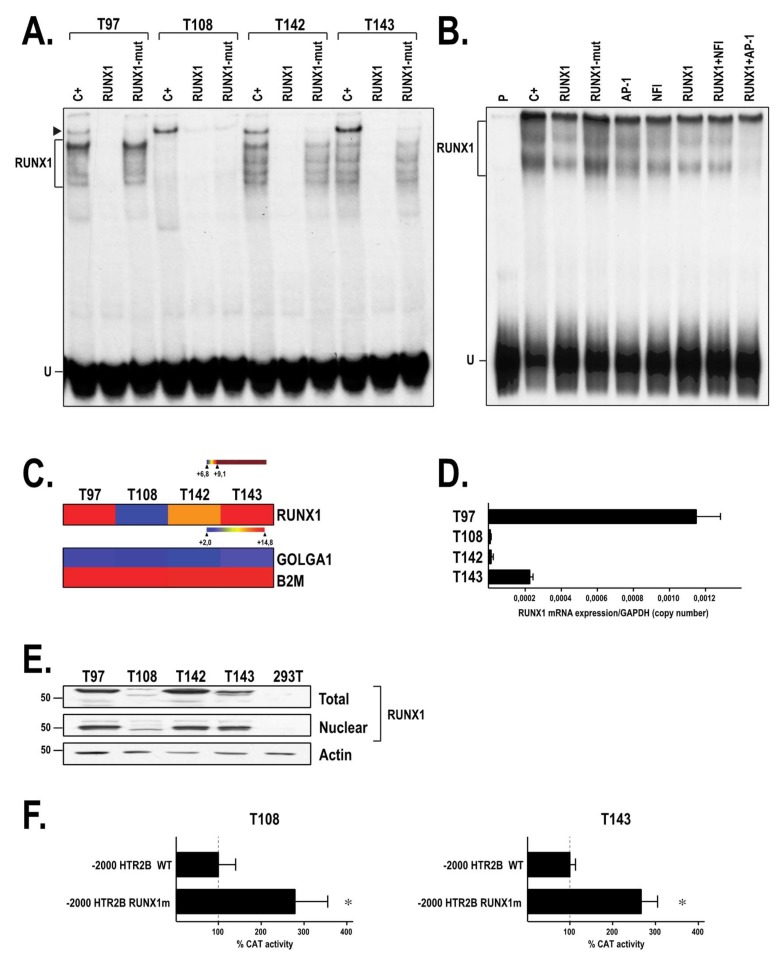
Expression of RUNX1 in the UM cell lines. (**A**) Nuclear proteins (20 µg) obtained from the UM cell lines T97, T108, T142 and T143 were incubated with a 5′ end-labeled oligonucleotide bearing the DNA sequence of the RUNX1 site identified in the *HTR2B* distal silencer, either alone (C+) or in the presence of a 500-fold molar excess of unlabeled RUNXI or its mutated derivative (RUNX1-mut). Formation of DNA-protein complexes was then monitored by EMSA. (**B**) Nuclear proteins from T143 cells were incubated with the 278 bp −1037/−1315 labeled probe bearing the distal silencer either alone (C+) or in the presence of a 100-fold molar excess of unlabeled RUNX1 or RUNX1-mut oligonucleotides, or with oligonucleotides bearing the target sites for NFI and AP-1. Expression of RUNX1 was also monitored at the transcriptional level in T97, T108, T142 and T143 cells by microarrays (panel (**C**)) and qPCR analyses (panel (**D**)). (**E**) Western blot analysis of RUNX1 in the UM cell lines T97, T108, T142 and T143. Actin expression was monitored as a normalization control. (**F**) CAT activities measured following transfection of the parental HTR2B/−2000 construct or its RUNX1-mutated derivative (−2000/RUNX1m(−1175)) in T97 and T143 UM cells. CAT activity is expressed relative to the level directed by the parental construct HTR2B/−2000. *: Values considered to be statistically significant from those obtained with wild-type HTR2B/−2000 (*P* value < 0.001).

As NFI does not appear to significantly contribute to the repressive influence mediated by the *HTR2B* distal silencer, we next exploited in vitro dimethylsulfate (DMS) methylation interference footprinting combined to EMSA in order to determine whether transcription factors other than NFI can bind this area from the *HTR2B* gene. As shown in Figure 7A, incubation of T143 nuclear proteins with the 5′-end labeled, DMS methylated 278 bp probe (from position −1037 to −1315) yielded three distinct DNA-protein complexes on gel (S1, S2 and S3). Interestingly, the methylated G residues that interfere with binding of the nuclear proteins yielding the S1, S2 and S3 complexes were the same (Gs at position −1125, −1126 and −1128) for each of them (Figure 7B). Detailed analysis of the DNA sequence bearing the protected G residues revealed that they were all located into one of the nine putative target sites identified for the transcription factor RUNX1 (Figure 2A and Supplementary Figure S1). We next derived a 28 bp, double-stranded oligonucleotide bearing the DNA sequence of the *HTR2B* RUNX1 site identified by DMS methylation interference and used it as a labeled probe in EMSA to monitor binding of this TF in nuclear extracts from our UM cell lines. Consistent with the EMSA results from Figure 7A, nuclear extracts from T97, T108, T142 and T143 cells all yielded multiple DNA-protein complexes when incubated with the RUNX1 labeled probe, although much weaker signals were observed with the extract from T108 cells (C+; Figure 8A). Addition of a 100-fold molar excess of unlabeled RUNX1 oligonucleotide entirely prevented the formation of the RUNX1 complexes whereas an unlabeled RUNX1 derivative (RUNX1-mut) in which the three G residues identified by DMS footprinting were mutated into adenines could not, thereby demonstrating the specificity for the formation of the multiple RUNX1 complexes seen on the EMSA. A DNA-protein complex of very low electrophoretic mobility on gel was also observed on the EMSA for all UM cell lines (arrowhead in Figure 8A). However, the protein yielding this complex could be competed both by the wild type and mutated RUNX1 oligonucleotides, indicating clearly that besides RUNX1, another nuclear protein expressed by all UM cells can bind the 28 bp RUNX1 probe.

Incubation of nuclear proteins from T143 cells with the 278 bp labeled probe from the *HTR2B* distal silencer that has been used for DMS footprinting also yielded the formation of multiple, diffuse complexes on gel (C+; Figure 8B). Addition of a 100-fold excess of unlabeled RUNX1 oligonucleotide reduced formation of the RUNX1 complexes by approximately 60%, whereas the mutated RUNX1-mut oligonucleotide had no impact. Interestingly, competing with unlabeled oligonucleotides bearing the target sites for either NFI or AP-1 also reduced formation of the RUNX1 DNA-protein complexes on gel (compare lanes 5 and 6 with lane 2; Figure 8B) suggesting that both NFI and AP-1 may contribute to the formation of the complexes seen in lane 2. Addition of both the RUNX1 and AP-1 competitors together in the reaction mix entirely prevented the formation of the DNA-protein complexes yielded by the T143 nuclear extract (compare lane 9 with lane 2). Analysis of RUNX1 mRNA expression by microarray (Figure 8C) and qPCR (Figure 8D) confirmed that T97 and T108 UM cells express the highest and lowest level of the RUNX1 transcript, respectively. Consistent with the EMSA results, the UM cell lines T97 and T142 were found, by Western blot analyses, to express high levels of the RUNX1 protein whereas T143 and T108 express moderate and low levels of that protein, respectively (Figure 8E). To determine the regulatory influence exerted by RUNX1 on the activity directed by the *HTR2B* gene promoter, we next transfected a derivative from HTR2B/−2000 that has its RUNX1 site mutated (−2000/RUNX1m) in both T108 and T143 UM cells. Disruption of the RUNX1 site resulted in a 2.8- and 2.7-fold increase in CAT activity in both T108 and T143 UM cells, respectively, relative to the level directed by the wild-type parental construct (Figure 8F) suggesting that RUNX1 functions as a repressor of *HTR2B* gene transcription in these cells.

### 2.5. Both RUNX1 and NFI Binds In Vivo to the HTR2B Promoter in Uveal Melanoma

Binding of NFI and RUNX1 to either the basal promoter or the distal silencer of the *HTR2B* gene was next examined in vivo by chromatin immunoprecipitation (ChIP)-qPCR analyses. The chromatin immunoprecipitated in each cell line by the NFI and RUNX1 antibodies was quantified by qPCR. As a negative control, the sonicated chromatin was also immunoprecipitated with an anti-IgG antibody. Data were normalized to both the input chromatin and the IgG signal. ChIP assays were performed on cross-linked chromatin isolated from the UM cell lines T97, T108, T142 and T143. The pairs of primers used for the qPCR analyses were selected to amplify the −123 to +83 (−9 NFI), −387 to −133 (−210 NFI), −1420 to −1229 (−1249 and −1275 NFI) and −1234 to −1022 (−1127 RUNX1) areas from the *HTR2B* gene (Supplementary Table S1). Consistent with the transfection results, a strong enrichment of the *HTR2B* basal promoter DNA segment was obtained when the chromatin was immunoprecipitated with the NFI antibody (Table 1). Indeed, the basal promoter area bearing the −9 NFI site was occupied by NFI in all four UM cell lines (enrichment of the amplified region in T97, T108, T142 and T143 was defined as “strong”, “weak”, “weak” and “very strong”, respectively). A similar scenario was also observed for the −210 NFI site (“weak”, “very strong”, “weak” and “strong” enrichment of the amplified region in T97, T108, T142 and T143, respectively; Table 1). The area bearing both the −1249 and −1275 NFI sites, which is also very close from the footprinted RUNX1 site (this area also bears two putative RUNX1 sites at positions −1280 and −1295), was ‘moderately’ bound by NFI in T97, T108 and T142 cells. Interestingly, and unlike the two previous promoter proximal areas that could not bind the RUNX1 TF in vivo, the −1420/−1229 area could efficiently bind RUNX1 in all but T108 UM cells (“strong”, “very weak”, “weak” and “weak” enrichment of the amplified region in T97, T108, T142 and T143, respectively; Table 1). Finally, binding of RUNX1 ranging from strong (in T142 and T143) to very strong (in T97) were observed in all but T108 UM cells when the area bearing the footprinted −1127 RUNX1 site was amplified using the −1234/−1022 specific primers (Table 1). Binding of NFI to this particular region could also be observed in T97 (strong), T108 (weak) and T143 (intermediate) cells. We conclude from these results that both NFI and RUNX1 cell-specifically interact in vivo with the promoter and 5′-flanking region of the *HTR2B* gene.

## 3. Discussion

Malignant uveal melanoma (UM) is the most common primary intraocular tumor in the adult population. Despite it being a rare disease (4 to 6 per million individuals), what makes UM so insidious is its propensity to remain dormant under the form of micro-metastases for as much as 12 to 15 years post-enucleation, before they progress into a clinically detectable metastatic disease. One particular characteristic that is clearly becoming a discriminating mark in order to identify those primary UM tumors at risk of progressing toward the metastatic disease is the detection of high levels of the *HTR2B* mRNA transcript [4,5,27]. However, no study has ever explored the mechanistic of this deregulated *HTR2B* gene expression at the transcription level. In this study, we investigated, both in vitro and in vivo, the molecular mechanisms that may contribute to the abnormally elevated expression of the *HTR2B* gene in UM. We demonstrated that *HTR2B* gene transcription is ensured in part by the binding of the transcription factors NFI and RUNX1 to distinct target sites within the basal promoter and upstream silencer elements. NFI was found to exert a strong positive regulatory influence on *HTR2B* gene expression, whereas RUNX1 acted as a weak repressor of that gene.

Transfection analyses revealed that alternating positive and negative regulatory regions are present along the *HTR2B* gene promoter and 5′-flanking region. Our search for putative target sites within these regulatory sequences revealed the presence of a particularly high number of target sites for the transcription factor NFI. This family of TFs comprises four isoforms (NFIA, -B, -C and -X) that have been reported to act either as activators or repressors of gene expression [28]. Microarrays, qPCR and Western blot analyses revealed that our UM cell lines express different combinations of NFI proteins that exert their regulatory influences by interacting with multiple target sites along the *HTR2B* gene regulatory sequences. In each of the gene systems we studied so far, NFI turned out to be a potent repressor of those target genes [20,21,22,29]. However, in the case of HTR2B, the members of this family clearly turned out to be activators of that gene, as site-directed mutation of both the −9 and −210 NFI sites almost completely abolishes transcription directed by the *HTR2B* promoter. Overexpression of the human NFI isoforms in T108 cells revealed that NFIA and NFIB acted positively on the transcription directed by the *HTR2B* gene promoter and 5′-flanking sequence, whereas both NFIC and NFIX had no impact under the conditions used. The stronger positive influence that both NFIA and NFIB exerted on the HTR2B/−2000 promoter activity (2.9- and 3.6-fold increase, respectively) relative to its impact on the HTR2B/−138 basal promoter construct (1.7- and 2.3-fold increases, respectively) is consistent with the presence of additional NFI sites along the −2000 HTR2B 5′-flanking sequence.

As stated above, T108 UM cells distinguishes themselves from the other cell lines by their clearly-reduced NFI binding capacity in EMSA (Figure 4A), which likely results from the absence of both the NFIB and NFIC isoforms (Figure 4C). However, the lack of NFIB and NFIC proteins in T108 UM cells does not correlate with their corresponding mRNA, which are abundantly expressed in these cells (Figure 2B). Discrepancies between expression of a specific gene at the mRNA and protein levels are common and many different mechanisms, such as protein phosphorylation, glycosylation or ubiquitinylation, may contribute these differences by altering the half-life of that protein. The NFI-C2 isoform (a splice variant of NFIC) has been reported to be subjected to proteosomal degradation through a Jak2-dependent tyrosine phosphorylation mechanism [30]. In addition, glycosylation of NFI has been postulated to delay its degradation by the proteasome in human skin keratinocytes [31]. Alternatively, it is well known that the protein product of some genes negatively regulates their own transcription through a negative feedback loop mechanism involving either the need for specific microRNA (miRNA) or activation of complex signal transduction pathways [32,33,34]. One particularly interesting aspect of our work was the demonstration that not all the NFI isoforms could bind to the distal silencer elements. Indeed, although all four recombinant NFI isoforms could bind with varying affinities to the consensus NFI site in EMSA, binding was only observed with the NFIC and NFIX isoforms when the NFI site-bearing distal silencer element is used as the labeled probe (Figure 5). This particularly interesting result suggests that besides their ability to recognize the prototypical NFI site [28,35], specific NFI isoforms likely possess the ability to discriminate amongst NFI degenerated DNA target sites.

As binding of NFI to the distal silencer apparently could not account for the negative regulatory influence directed by this element, we kept searching for other TFs that may repress *HTR2B* gene expression by interacting within the *HTR2B* distal silencer. The use of both EMSA and DMS methylation interference footprinting allowed us to demonstrate the interaction of RUNX1, a transcription factor widely expressed in hematopoietic cells and indispensable for the establishment of definitive hematopoiesis, to a target site (at position −1127) located within the distal silencer. That DNA-protein complexes with very distinctive electrophoretic mobilities on gel all protected the same G residues in DMS methylation interference footprinting suggest that a single protein (for instance RUNX1) establishes contact with a specific target site in the *HTR2B* distal silencer (at position −1127) and that its interaction with other nuclear proteins (through protein-protein interactions) reduces its electrophoretic mobility to yield the multiple band pattern seen in the EMSA (Figure 7A and Figure 8A,B). RUNX1 has been reported to physically interact, or synergize with a variety of other factors, such as E26 Transformation-specific transcription factor-1 (Ets-1) [36], C/EBP [37], the MYB proto-oncogene (c-Myb) [38], and most interestingly, AP-1 [39,40]. This is consistent with the results from the EMSA shown in Figure 8 that clearly suggests the cooperative binding of both the RUNX1 and AP-1 transcription factors to/or nearby the −1127 RUNX1 target site identified in the *HTR2B* distal silencer. Indeed, three putative AP-1 target sites with a 6-on-7 nucleotide match to the AP-1 consensus site (5′-TGAG/CTCA-3′) were identified nearby the RUNX1 site in the *HTR2B* distal silencer at positions −1225/−1219 (5′-TGcCTCA-3′), −1074/−1068 (5′-aGACTCA-3′) and −1021/−1015 (5′-TGAGcCA-3′), the closest being located at only 49 nucleotides downstream from the RUNX1 site. RUNX1 has also been reported to bind either as a monomer or a dimer to the colony stimulating factor 2 (GM-CSF) enhancer, which also causes the formation of multiple bands on the EMSA [41]. Besides AP-1, many RUNX1-regulated genes have been reported to be co-occupied also by the TF class I myosin (MyoD) during muscle regeneration [42]. It is interesting to point out that low to high expression of MyoD is observed in our UM cell-lines and that two putative target sites for MyoD have been identified 65 bp downstream from the −1127 RUNX1 site (Supplementary Figure S1).

We can’t rule out the possibility that RUNX1 may interact with other, yet uncharacterized target sites along the 5′ flanking region of the *HTR2B* gene, as other such sites have been identified using the TFSEARCH program. Two of these degenerated RUNX1 sites have been identified in the basal promoter region (at positions −86 and −205). However, neither are likely functional at binding RUNX1, as none of the regions bearing these two sites could be efficiently enriched in quantitative chromatin immunoprecipitation (qChIP) in vivo analyses (Table 1; promoter regions −123/+83 and −387/−133). Furthermore, we demonstrated that the area from the *HTR2B* gene that also contains the −1127 RUNX1 site footprinted in the present study was dramatically enriched by this TF in all but T108 UM cells (Table 1; promoter region −1234/−1022), which is consistent with the reduced expression of this TF at the protein level observed in this cell line (Figure 8E). Interestingly, the area bearing both the −1249 and −1275 NFI sites located immediately upstream of that containing the −1127 footprinted RUNX1 site was considerably enriched by immunoprecipitation of RUNX1 in qChIP analyses in all but T108 UM cells (Table 1; promoter region −1420/−1229). Interestingly, two additional RUNX1 putative target sites were also identified upstream of the site footprinted in this study, at positions −1278 and −1290. Our qChIP results therefore suggest that besides the −1127 site, RUNX1 may also bind to one (or both) of these additional target sites in vivo.

Identifying RUNX1 as a regulator of *HTR2B* gene expression in UM cancer cells is particularly relevant to our study in that deregulation of this TFs appears to be a characteristic of many types of cancers, including acute leukemia (reviewed in [43]), human colorectal [44], prostate [45], endometrial, ovarian [46], and human skin and oral squamous cell carcinomas [47]. Interestingly, Recouvreux et al. have recently demonstrated a physical interaction between the tumor suppressor Foxp3 and RUNX1 that suppresses the trans-activating properties of RUNX1 and suggested that any disruption in this equilibrium in favor of RUNX1 (which is viewed as a tumor enhancer based on their results) may contribute to breast cancer development [48]. It should prove interesting to examine if Foxp3 is also expressed in UM cells and define whether the multi-band pattern observed in the EMSA for RUNX1 may also be accounted for, at least in part, by a direct physical interaction between RUNX1 and Foxp3.

## 4. Materials and Methods

This study was conducted in agreement with the Helsinki declaration and was performed under the guidelines of the research ethics committee of the “CHU de Québec” (ethic code: DR-002-1305, protocol renewal approved on 30 January 2018).

### 4.1. Cell Culture

The UM cell lines T97, T98, T108, T111, T128, T131, T132, T142, T143, T151 and T157 were each cultured from the primary tumors of different patients diagnosed with this type of cancer and many of them have been previously described [49,50,51,52]. Human embryonic kidney 293T cells were obtained from ATCC (ATCC CRL-3216; Manassas, VA, USA). All cells were cultured in Dulbecco/Vogt modified Eagle’s minimal essential medium (DMEM) Multicell (high glucose, with l-glut, without L-Pyruvate; Wisent, Québec, QC, Canada) supplemented with 10% fetal bovine serum (FBS) High quality (Wisent, Québec, QC, Canada) and 0.002% *v*/*v* gentamicin (Life Technologies (distributed by Thermo Fisher Scientific Inc., Rockford, IL, USA) at 37 °C under 5% CO_2_.

### 4.2. Indirect Immunofluorescence

Indirect immunofluorescence assays were performed on UM cell lines (T97, T108, T142 and T143) grown on glass coverslips and acetone-fixed (10 min at −20 °C). Cells were incubated for 45 min with a primary antibody directed against the HTR2B protein (SAB4501476, Sigma-Aldrich, Oakville, ON, Canada) and used at an optimal dilution of 1:100 in phosphate-buffered saline (137 mM NaCl, 2.7 mM KCl, 6.5 mM Na_2_HPO_4_ and 1.5 mM KH_2_PO_4_) containing 1% bovine serum albumin. The secondary antibody (rabbit anti-mouse IgG (H + L) conjugated with Alexa-fluor^®^ 488 (1:400; Molecular Probes, Burlington, ON, Canada)) was incubated for 30 min. Cell nuclei were revealed following Hoechst reagent 33258 labeling (1:100; Sigma-Aldrich). They were observed with an epifluorescence microscope (Zeiss Imager.Z2; Zeiss Canada Ltd., North York, ON, Canada) and photographed (AxioVision software, Carl Zeiss Microscopy, Peabody, MA, USA).

### 4.3. Plasmid Constructs and Oligonucleotides

The details regarding the production of the different recombinant plasmids used in this study are described in Supplementary Materials. The double-stranded oligonucleotides used either as labeled probe or unlabeled competitors in the EMSAs were chemically synthesized using a Biosearch 8700 apparatus (Integrated DNA Technologies, Inc., Coralville, WA, USA). Their DNA sequences are listed in Supplementary Table S1.

### 4.4. Expression of the Human Recombinant NFI Isoforms

The details regarding the cloning, expression and purification of each of the NFI isoforms used in this study are described in Supplementary Materials.

### 4.5. Transient Transfections and CAT Assays

All HTR2B/CAT recombinant plasmids were transiently transfected into the UM cell lines T97, T108, T142 and T143 grown to sub-confluence (70% coverage of the culture plate) into 6-wells tissue-culture plates using either the polycationic detergent Lipofectamine 2000 (Life Technologies, Burlington, ON, Canada) [53] or the K2^®^ Transfection System following the manufacturer’s instructions (BIONTEX, München, Germany). Each tissue-culture well received 1 µg of the test plasmid and 0.5 µg of the hGH-encoding plasmid PXGH5, except for T143 cells, that were transfected with 2 µg of the test plasmid and 0.5 µg of PXGH5. When indicated, high levels NFI expressing pLenti6V5A derivatives (0.5 µg) were co-transfected along with the HTR2B/CAT constructs (0.5 µg) in T108 cells. All cells were harvested 48 h following transfection and CAT activities were determined and normalized to the hGH secreted in the medium as previously described [54].

### 4.6. Preparation of Nuclear Extracts and EMSA

All tissue-cultured cells were grown to mid-confluence (70% coverage of the culture flasks) prior to preparation of nuclear extracts and their use in EMSAs following previously described procedures [55,56]. Briefly, EMSAs were carried out by incubating nuclear proteins with a 5′ ^32^P-end-labelled BglII/BstXI, 278 bp DNA fragment bearing most of the *HTR2B* distal silencer from position -1315 (BstXI site) to −1037 (BglII site) and labeled at its BglII site. When indicated, a 238 bp XbaI/SacI DNA fragment bearing the entire basal promoter of the *HTR2B* gene, or double-stranded oligonucleotides bearing either the high affinity binding site for the transcription factor NFI [57] or the RUNX1 binding site identified in the *HTR2B* distal silencer were also 5′ ^32^P-end-labeled and used as probes in EMSAs. Briefly, 5 × 10^4^ cpm labeled probe was incubated with the amount of nuclear proteins specified in the legend of each figure in the presence of 2 μg of poly(dI:dC) (Amersham Biosciences, Piscataway, NJ, USA) and 50 mM KCl in buffer D [10 mM Hepes pH 7.9, 10% *v*/*v* glycerol, 0.1 mM EDTA, 0.5 mM DTT (dithiothreitol; Sigma-Aldrich Canada, Oakville, ON, Canada) and 0.25 mM phenylmethylsulfonyl fluoride (PMSF; Sigma-Aldrich Canada)]. Synthetic oligomers bearing the recognition sequence for known TFs (Supplementary Table S1) were added as unlabeled competitors (10- to 750-fold molar excesses, as specified in the figure’s legends) during the assay. DNA–protein complexes were next separated by gel electrophoresis through either 6% (for both the 278- and 238 bp *HTR2B* labeled probe) or 8% (for both the NFI and RUNX1 probes) non-denaturing polyacrylamide gels run against Tris-glycine buffer (50 mM Tris, 2.5 mM EDTA, 0.4 M glycine) at 4 °C. Position of the DNA–protein complexes was then revealed upon autoradiography of the dried gels at −80 °C. Super-shift experiments were conducted by incubating 5 µg nuclear proteins from T143 UM cells in the presence of 2 µL of a polyclonal antibody raised against NFI (SC-5567; Santa Cruz Biotechnology, Inc., Dallas, TX, USA).

### 4.7. Methylation Interference Footprinting

The BglII/BstXI 278 bp *HTR2B* distal silencer fragment used in the EMA was 5′ end-labeled at its BglII site and partially methylated with DMS as described [58]. Thirty µg nuclear proteins from T143 UM cells were then incubated with 5 × 10^5^ cpm of the methylated probe together with 5 µg poly(dI:dC) in buffer D for 5 min at room temperature prior to separation of DNA-protein complexes on a 6% native polyacrylamide gel run against Tris-glycine buffer. The complexes formed were isolated by electroelution upon their visualization by autoradiography as previously described [59]. The isolated labeled DNA was then treated with piperidine and further analyzed on an 8% sequencing gel as described [58].

### 4.8. Western Blots

Western blots were conducted as described [53,60,61] using antibodies directed against the following proteins: total NFI (sc-5567 (polyclonal), 1:2000; Santa Cruz Biotechnology), NFI-A (ab11988 (polyclonal), 1:500; Abcam, Toronto, ON, Canada), NFI-B (ab51352-100 (monoclonal), 1:100; Abcam), NFI-C (ab89516 (monoclonal), 1:500; Abcam), NFI-X (ab67169 (polyclonal), 1:100; Abcam), RUNX1 (ab23980, 1:1000; Abcam) and a peroxidase-conjugated AffiniPure Goat secondary antibody against mouse IgG (1:2500 dilution; Jackson ImmunoResearch Laboratories, West Grove, PA, USA).

### 4.9. Gene Expression Profiling

Isolation of total RNA and microarray analysis, which all comply with the Minimum Information About a Microarray Experiment (MIAME) requirements, were conducted as recently reported [62]. Data have been deposited in NCBIs Gene Expression Omnibus (GEO, available online: http://www.ncbi.nlm.nih.gov/geo/) and are accessible through GEO Series accession number GSE GSE86915.

### 4.10. Chromatin Immunoprecipitation Assays (ChIP)-qPCR

Description of the detailed experimental procedure for conducting in vivo ChIP-qPCR can be found in Supplementary Materials.

### 4.11. Quantitative PCR (qPCR)

Quantity and quality of total RNA from all UM cell lines was assessed using an Agilent Technologies 2100 bioanalyzer and RNA 6000 Nano LabChip kit (Agilent Technologies, Santa Clara, CA, USA). RNAs were used for qPCR and gene profiling analyses only if their RNA integrity number (RIN) was greater than 7 over 10. Reverse transcription was performed using the High Capacity cDNA Reverse Transcrition Kit from AB applied biosystems (Foster City, CA, USA) following the manufacturer’s instructions. Equal amounts of cDNA were run in quadruplicate and amplified in a 20 µL reaction containing 10 µL of 2× SYBR green Advanced qPCR mastermix (Wisent, Québec, QC, Canada), 100 nM of upstream and downstream primers, and 1 ng of cDNA target. No-template controls were also used as recommended. The mixture was incubated at 95 °C for 3 min, and then cycled at 95 °C for 10 s and at 60 °C for 20 s for 35 cycles using the QIAGEN Rotor-Gene Q real-time cycler. Amplification efficiencies were validated and normalized to the actin mRNA transcript and quantity of target genes were calculated according to a standard curve. The primers were designed using PrimerQuest Tool (IDT, Integrated DNA technologies, Coralville, WA, USA) and are listed in Supplementary Table S1.

### 4.12. Statistical Analyses

Student’s *t*-test was performed for comparison of the groups in both transfection and qRT-PCR analyses. Differences were considered to be statistically significant at *P* < 0.05. All data are also expressed as mean ± SD.

## Figures and Tables

**Table 1:** In vivo detection of NFI and RUNX1 binding to the *HTR2B* promoter by ChIP-qPCR.

UM Cell Line	T97		T108		T142		T143	
Immunoprecipitated TF *	NFI	RUNX1	NFI	RUNX1	NFI	RUNX1	NFI	RUNX1
*HTR2B* region								
−123/+83 (−9 NFI)	2,766,666	158	1082	8	5260	6	554,945,054	51
387/−133 (−210 NFI)	19,814	43	14,938,271	93	1682	515	5,586,956	63
−1420/−1229 (−1249/−1275 NFI)	3533	1,596,666	132,564	4	28,220	8458	12	1713
−1234/−1022 (−1127 RUNX1)	1,882,926	760,975,609	1067	1	66	2,368,750	34,982	1,269,624

Data are presented as fold changes of NFI or RUNX1 enrichment (relative to input chromatin) over IgG enrichment (relative to input chromatin). *: × 10^−10^.

## Supplementary Materials

Supplementary materials can be found online at http://www.mdpi.com/1422-0067/19/10/3272/s1.

## References

[1] Singh A.D., Topham A. (2003). Incidence of uveal melanoma in the United States: 1973–1997. Ophthalmology.

[2] Kujala E., Makitie T., Kivela T. (2003). Very long-term prognosis of patients with malignant uveal melanoma. Investig. Ophthalmol. Vis. Sci..

[3] Onken M.D., Worley L.A., Ehlers J.P., Harbour J.W. (2004). Gene expression profiling in uveal melanoma reveals two molecular classes and predicts metastatic death. Cancer Res..

[4] Onken M.D., Worley L.A., Tuscan M.D., Harbour J.W. (2010). An accurate, clinically feasible multi-gene expression assay for predicting metastasis in uveal melanoma. J. Mol. Diagn..

[5] Zhang Y., Yang Y., Chen L., Zhang J. (2014). Expression analysis of genes and pathways associated with liver metastases of the uveal melanoma. BMC Med. Genet..

[6] Raymond J.R., Mukhin Y.V., Gelasco A., Turner J., Collinsworth G., Gettys T.W., Grewal J.S., Garnovskaya M.N. (2001). Multiplicity of mechanisms of serotonin receptor signal transduction. Pharmacol. Ther..

[7] Harbour J.W., Onken M.D., Roberson E.D., Duan S., Cao L., Worley L.A., Council M.L., Matatall K.A., Helms C., Bowcock A.M. (2010). Frequent mutation of BAP1 in metastasizing uveal melanomas. Science.

[8] Van Raamsdonk C.D., Bezrookove V., Green G., Bauer J., Gaugler L., O’Brien J.M., Simpson E.M., Barsh G.S., Bastian B.C. (2009). Frequent somatic mutations of GNAQ in uveal melanoma and blue naevi. Nature.

[9] Banes A.K., Shaw S.M., Tawfik A., Patel B.P., Ogbi S., Fulton D., Marrero M.B. (2005). Activation of the JAK/STAT pathway in vascular smooth muscle by serotonin. Am. J. Physiol. Cell Physiol..

[10] Naito K., Tanaka C., Mitsuhashi M., Moteki H., Kimura M., Natsume H., Ogihara M. (2016). Signal Transduction Mechanism for Serotonin 5-HT2B Receptor-Mediated DNA Synthesis and Proliferation in Primary Cultures of Adult Rat Hepatocytes. Biol. Pharmaceut. Bull..

[11] Choi D.S., Ward S.J., Messaddeq N., Launay J.M., Maroteaux L. (1997). 5-HT2B receptor-mediated serotonin morphogenetic functions in mouse cranial neural crest and myocardiac cells. Development.

[12] Vicaut E., Laemmel E., Stucker O. (2000). Impact of serotonin on tumour growth. Ann. Med..

[13] Siddiqui E.J., Thompson C.S., Mikhailidis D.P., Mumtaz F.H. (2005). The role of serotonin in tumour growth (review). Oncol. Rep..

[14] Svejda B., Kidd M., Giovinazzo F., Eltawil K., Gustafsson B.I., Pfragner R., Modlin I.M. (2010). The 5-HT(2B) receptor plays a key regulatory role in both neuroendocrine tumor cell proliferation and the modulation of the fibroblast component of the neoplastic microenvironment. Cancer.

[15] Soll C., Jang J.H., Riener M.O., Moritz W., Wild P.J., Graf R., Clavien P.A. (2010). Serotonin promotes tumor growth in human hepatocellular cancer. Hepatology.

[16] Fernandez-Ranvier G.G., Weng J., Yeh R.F., Khanafshar E., Suh I., Barker C., Duh Q.Y., Clark O.H., Kebebew E. (2008). Identification of biomarkers of adrenocortical carcinoma using genomewide gene expression profiling. Arch. Surg..

[17] Dizeyi N., Bjartell A., Hedlund P., Tasken K.A., Gadaleanu V., Abrahamsson P.A. (2005). Expression of serotonin receptors 2B and 4 in human prostate cancer tissue and effects of their antagonists on prostate cancer cell lines. Eur. Urol..

[18] Henriksen R., Dizeyi N., Abrahamsson P.A. (2012). Expression of serotonin receptors 5-HT1A, 5-HT1B, 5-HT2B and 5-HT4 in ovary and in ovarian tumours. AntiCancer Res..

[19] Reisoli E., De Lucchini S., Anelli T., Biagioni S., Nardi I., Ori M. (2008). Overexpression of 5-HT2B receptor results in retinal dysplasia and defective ocular morphogenesis in Xenopus embryos. Brain Res..

[20] Gaudreault M., Vigneault F., Gingras M.E., Leclerc S., Carrier P., Germain L., Guerin S.L. (2008). Transcriptional regulation of the human alpha6 integrin gene by the transcription factor NFI during corneal wound healing. Investig. Ophthalmol. Vis. Sci..

[21] Gingras M.E., Masson-Gadais B., Zaniolo K., Leclerc S., Drouin R., Germain L., Guerin S.L. (2009). Differential binding of the transcription factors Sp1, AP-1, and NFI to the promoter of the human alpha5 integrin gene dictates its transcriptional activity. Investig. Ophthalmol. Vis. Sci..

[22] Laniel M.A., Poirier G.G., Guerin S.L. (2001). Nuclear factor 1 interferes with Sp1 binding through a composite element on the rat poly(ADP-ribose) polymerase promoter to modulate its activity in vitro. J. Biol. Chem..

[23] Brettingham-Moore K.H., Taberlay P.C., Holloway A.F. (2015). Interplay between Transcription Factors and the Epigenome: Insight from the Role of RUNX1 in Leukemia. Front. Immunol..

[24] Kuvardina O.N., Herglotz J., Kolodziej S., Kohrs N., Herkt S., Wojcik B., Oellerich T., Corso J., Behrens K., Kumar A. (2015). RUNX1 represses the erythroid gene expression program during megakaryocytic differentiation. Blood.

[25] Roulet E., Bucher P., Schneider R., Wingender E., Dusserre Y., Werner T., Mermod N. (2000). Experimental analysis and computer prediction of CTF/NFI transcription factor DNA binding sites. J. Mol. Biol..

[26] Roulet E., Busso S., Camargo A.A., Simpson A.J., Mermod N., Bucher P. (2002). High-throughput SELEX SAGE method for quantitative modeling of transcription-factor binding sites. Nat. Biotechnol..

[27] Malho P., Dunn K., Donaldson D., Dubielzig R.R., Birand Z., Starkey M. (2013). Investigation of prognostic indicators for human uveal melanoma as biomarkers of canine uveal melanoma metastasis. J. Small Anim. Pract..

[28] Gronostajski R.M. (2000). Roles of the NFI/CTF gene family in transcription and development. Gene.

[29] Steffensen K.R., Holter E., Tobin K.A., Leclerc S., Gustafsson J.A., Guerin S.L., Eskild W. (2001). Members of the nuclear factor 1 family reduce the transcriptional potential of the nuclear receptor LXRα promoter. Biochem. Biophys. Res. Commun..

[30] Nilsson J., Bjursell G., Kannius-Janson M. (2006). Nuclear Jak2 and transcription factor NF1-C2: A novel mechanism of prolactin signaling in mammary epithelial cells. Mol. Cell. Biol..

[31] Duval C., Gaudreault M., Vigneault F., Touzel-Deschenes L., Rochette P.J., Masson-Gadais B., Germain L., Guerin S.L. (2012). Rescue of the transcription factors Sp1 and NFI in human skin keratinocytes through a feeder-layer-dependent suppression of the proteasome activity. J. Mol. Biol..

[32] Xia S.S., Zhang G.J., Liu Z.L., Tian H.P., He Y., Meng C.Y., Li L.F., Wang Z.W., Zhou T. (2017). MicroRNA-22 suppresses the growth, migration and invasion of colorectal cancer cells through a Sp1 negative feedback loop. Oncotarget.

[33] Ren D., Wang M., Guo W., Huang S., Wang Z., Zhao X., Du H., Song L., Peng X. (2014). Double-negative feedback loop between ZEB2 and miR-145 regulates epithelial-mesenchymal transition and stem cell properties in prostate cancer cells. Cell Tissue Res..

[34] Ohno Y., Saeki K., Yasunaga S., Kurogi T., Suzuki-Takedachi K., Shirai M., Mihara K., Yoshida K., Voncken J.W., Ohtsubo M. (2014). Transcription of the Geminin gene is regulated by a negative-feedback loop. Mol. Biol. Cell.

[35] Murtagh J., Martin F., Gronostajski R.M. (2003). The Nuclear Factor I (NFI) gene family in mammary gland development and function. J. Mammary Gland Biol. Neoplasia.

[36] Kim W.Y., Sieweke M., Ogawa E., Wee H.J., Englmeier U., Graf T., Ito Y. (1999). Mutual activation of Ets-1 and AML1 DNA binding by direct interaction of their autoinhibitory domains. EMBO J..

[37] Zhang D.E., Hetherington C.J., Meyers S., Rhoades K.L., Larson C.J., Chen H.M., Hiebert S.W., Tenen D.G. (1996). CCAAT enhancer-binding protein (C/EBP) and AML1 (CBF alpha2) synergistically activate the macrophage colony-stimulating factor receptor promoter. Mol. Cell. Biol..

[38] Hernandez-Munain C., Krangel M.S. (1995). c-Myb and core-binding factor/PEBP2 display functional synergy but bind independently to adjacent sites in the T-cell receptor delta enhancer. Mol. Cell. Biol..

[39] Pencovich N., Jaschek R., Tanay A., Groner Y. (2011). Dynamic combinatorial interactions of RUNX1 and cooperating partners regulates megakaryocytic differentiation in cell line models. Blood.

[40] Bertrand-Philippe M., Ruddell R.G., Arthur M.J., Thomas J., Mungalsingh N., Mann D.A. (2004). Regulation of tissue inhibitor of metalloproteinase 1 gene transcription by RUNX1 and RUNX2. J. Biol. Chem..

[41] Bowers S.R., Calero-Nieto F.J., Valeaux S., Fernandez-Fuentes N., Cockerill P.N. (2010). Runx1 binds as a dimeric complex to overlapping Runx1 sites within a palindromic element in the human GM-CSF enhancer. Nucleic Acids Res..

[42] Umansky K.B., Gruenbaum-Cohen Y., Tsoory M., Feldmesser E., Goldenberg D., Brenner O., Groner Y. (2015). Runx1 Transcription Factor Is Required for Myoblasts Proliferation during Muscle Regeneration. PLoS Genet..

[43] Ichikawa M., Yoshimi A., Nakagawa M., Nishimoto N., Watanabe-Okochi N., Kurokawa M. (2013). A role for RUNX1 in hematopoiesis and myeloid leukemia. Int. J. Hematol..

[44] Slattery M.L., Lundgreen A., Herrick J.S., Caan B.J., Potter J.D., Wolff R.K. (2011). Associations between genetic variation in RUNX1, RUNX2, RUNX3, MAPK1 and eIF4E and riskof colon and rectal cancer: Additional support for a TGF-beta-signaling pathway. Carcinogenesis.

[45] Huang S.P., Lan Y.H., Lu T.L., Pao J.B., Chang T.Y., Lee H.Z., Yang W.H., Hsieh C.J., Chen L.M., Huang L.C. (2011). Clinical significance of runt-related transcription factor 1 polymorphism in prostate cancer. BJU Int..

[46] Planaguma J., Gonzalez M., Doll A., Monge M., Gil-Moreno A., Baro T., Garcia A., Xercavins J., Alameda F., Abal M. (2006). The up-regulation profiles of p21WAF1/CIP1 and RUNX1/AML1 correlate with myometrial infiltration in endometrioid endometrial carcinoma. Hum. Pathol..

[47] Scheitz C.J., Lee T.S., McDermitt D.J., Tumbar T. (2012). Defining a tissue stem cell-driven Runx1/Stat3 signalling axis in epithelial cancer. EMBO J..

[48] Recouvreux M.S., Grasso E.N., Echeverria P.C., Rocha-Viegas L., Castilla L.H., Schere-Levy C., Tocci J.M., Kordon E.C., Rubinstein N. (2016). RUNX1 and FOXP3 interplay regulates expression of breast cancer related genes. Oncotarget.

[49] Duval C., Zaniolo K., Leclerc S., Salesse C., Guerin S.L. (2015). Characterization of the human α9 integrin subunit gene: Promoter analysis and transcriptional regulation in ocular cells. Exp. Eye Res..

[50] Landreville S., Vigneault F., Bergeron M.A., Leclerc S., Gaudreault M., Morcos M., Mouriaux F., Salesse C., Guerin S.L. (2011). Suppression of α5 gene expression is closely related to the tumorigenic properties of uveal melanoma cell lines. Pigment Cell Melanoma Res..

[51] Molloy-Simard V., St-Laurent J.F., Vigneault F., Gaudreault M., Dargis N., Guerin M.C., Leclerc S., Morcos M., Black D., Molgat Y. (2012). Altered expression of the poly(ADP-ribosyl)ation enzymes in uveal melanoma and regulation of PARG gene expression by the transcription factor ERM. Investig. Ophthalmol. Vis. Sci..

[52] Mouriaux F., Zaniolo K., Bergeron M.A., Weidmann C., De La Fouchardiere A., Fournier F., Droit A., Morcos M.W., Landreville S., Guerin S.L. (2016). Effects of Long-term Serial Passaging on the Characteristics and Properties of Cell Lines Derived From Uveal Melanoma Primary Tumors. Investig. Ophthalmol. Vis. Sci..

[53] Larouche K., Leclerc S., Salesse C., Guerin S.L. (2000). Expression of the alpha 5 integrin subunit gene promoter is positively regulated by the extracellular matrix component fibronectin through the transcription factor Sp1 in corneal epithelial cells in vitro. J. Biol. Chem..

[54] Pothier F., Ouellet M., Julien J.P., Guerin S.L. (1992). An improved CAT assay for promoter analysis in either transgenic mice or tissue culture cells. DNA Cell Biol..

[55] Roy R.J., Gosselin P., Guerin S.L. (1991). A short protocol for micro-purification of nuclear proteins from whole animal tissue. Biotechniques.

[56] Gaudreault M., Gingras M.E., Lessard M., Leclerc S., Guerin S.L. (2009). Electrophoretic mobility shift assays for the analysis of DNA-protein interactions. Methods Mol. Biol..

[57] De Vries E., van Driel W., van den Heuvel S.J., van der Vliet P.C. (1987). Contactpoint analysis of the HeLa nuclear factor I recognition site reveals symmetrical binding at one side of the DNA helix. EMBO J..

[58] Baldwin A.S.J., Struhl K. (1989). Current Protocols in Molecular Biology. Current Protocols in Molecular Biology.

[59] Harvey M., Brisson I., Guerin S.L. (1993). A simple apparatus for fast and inexpensive recovery of DNA from polyacrylamide gels. Biotechniques.

[60] Gaudreault M., Vigneault F., Leclerc S., Guerin S.L. (2007). Laminin reduces expression of the human alpha6 integrin subunit gene by altering the level of the transcription factors Sp1 and Sp3. Investig. Ophthalmol. Vis. Sci..

[61] Gingras M.E., Larouche K., Larouche N., Leclerc S., Salesse C., Guerin S.L. (2003). Regulation of the integrin subunit alpha5 gene promoter by the transcription factors Sp1/Sp3 is influenced by the cell density in rabbit corneal epithelial cells. Investig. Ophthalmol. Vis. Sci..

[62] Couture C., Zaniolo K., Carrier P., Lake J., Patenaude J., Germain L., Guerin S.L. (2016). The tissue-engineered human cornea as a model to study expression of matrix metalloproteinases during corneal wound healing. Biomaterials.

