# Supplementary material for: Transcription of the Human 5-Hydroxytryptamine Receptor 2B (HTR2B) Gene Is under the Regulatory Influence of the Transcription Factors NFI and RUNX1 in Human Uveal Melanoma
Source: Int J Mol Sci. 2018 Oct 21;19(10):3272. doi: 10.3390/ijms19103272 (PMC6214142; doi:10.3390/ijms19103272)
Supplement: Supplementary file 1 [file ijms-19-03272-s001.pdf]

SUPPLEMENTARY MATERIAL

Supplementary Methods

Plasmid constructs

A near 2 kb fragment bearing the 5' flanking sequence of the human *HTR2B* gene (from position -2000 to +96 relative to the theoretical mRNA start site) was synthesized and cloned by *Blue Heron* (Bothell, WA, USA) upstream of the CAT reporter gene into the pCATBasic vector (Promega, Madison, WI, USA). Derivatives from the -2000/HTR2B construct bearing various deletions of the *HTR2B* promoter were then produced by first digesting the parental plasmid with the restriction enzyme SbfI (target site located 5' into the multiple cloning site (MCS) of pCATBasic) followed by a second digestion with one of the following enzymes: SpeI (cuts at position -1297), NsiI (cuts at -710), StuI (cuts at -430) or SacI (cuts at -138). The restriction site overhangs of the double-digested plasmids were blunt ended by treatment with Klenow (New England Biolabs Whitby, ON, Canada) and ligated using T4 DNA ligase (New England Biolabs). All recombinant *HTR2B*/CAT plasmids therefore share the same 3' end (at position +96) but different 5' termini (5' positions: -2000, -1297, -710, -430 -138). Target sites for the transcription factor NFI identified at position -9, -210, -1249 and -1275 as well as that for the transcription factor RUNX1 at position -1134 were mutated using the *QuikChange Lightning Multi Site-Directed Mutagenesis Kit* from Agilent Technologies (Santa Clara, CA, USA) according to manufacturer's instructions.

Construction of the pLenti6V5A derivatives that express high levels of each of the four human NFI isoforms (NFIA, -B, -C and –X) have been recently described [1].

Expression of the human recombinant NFI isoforms

Expression and purification of each of the NFI isoforms was performed using the IMPACT (*Intein Mediated Purification with an Affinity Chitin-binding Tag*) protein purification system as recommended by the supplier (New England Biolabs). cDNAs encoding each of the NFI isoforms were cloned in the plasmid pTXB1 and then transformed in *E. coli* ER2566 cells. Bacterially produced NFI proteins were then bound to the chitin resin, washed with 100 ml of lysis buffer (20 mM Na-HEPES pH8.5, 500 mM NaCl, 1 mM EDTA, 0.1% Triton X-100), incubated overnight in 1 column volume of lysis buffer containing 50 mM DTT. NFI proteins were then collected into 2 fractions of 500µl. The proteins remaining on the column were collected following the addition of 5 ml of DTT-free lysis buffer. All collected fractions were dialyzed against DNAseI buffer A (50 mM KCl, 20 mM K_3PO_4 pH 7.4, 1 mM $MgCl_2$, 1 mM β-mercaptoethanol, 20% glycerol) and kept frozen at -80°C. When needed, 30

μl of dialyzed, recombinant NFI proteins were phosphorylated for 1 h at 30°C with casein kinase II (500 U; New England Biolabs) in CKII buffer containing 19 μM ATP (final volume of 75 μl) prior to their use in EMSA.

Chromatin immunoprecipitation assays (ChIP)-qPCR

ChIP analyses were conducted using the Zymo-SpinTM ChIP kit (Zymo Research, Irvine, CA, USA) on the UM cell lines T97, T108, T142 and T143. Briefly, when they reached 80% confluence on 150-mm tissue culture dishes, UM cells were harvested and $5x10^6$ cells were cross-linked with 1% formaldehyde for 7 minutes prior to sonication of cross-linked chromatins. The average size of the sonicated DNA ranged between 300-600 bp. Cross-linked chromatin was then immunoprecipitated with 1 μg antibodies against the transcription factors RUNX1 or NFI as previously reported [2, 3]. Incubation was also performed with a mouse antibody against IgG2a Fc (Chemicon, Temecula, CA) as a negative control. qPCR analyses were then performed using the specific primers listed in Supplementary Table 1. The values for the samples immunoprecipitated by the anti-NFI, anti-RUNX1 and control IgG were normalized both to the input chromatin and the IgG signal. ChIP results were confirmed by two independent experiments and qPCR was performed in quadruplicate for each sample. As a negative control, each ChIP sample was also subjected to qPCR using primers (p21-F and p21-R; Supplementary Table 1) specific to a region located ~2 Kbp upstream from the human p21 promoter (cycle parameters are described in the next sub-section).

Supplementary figures

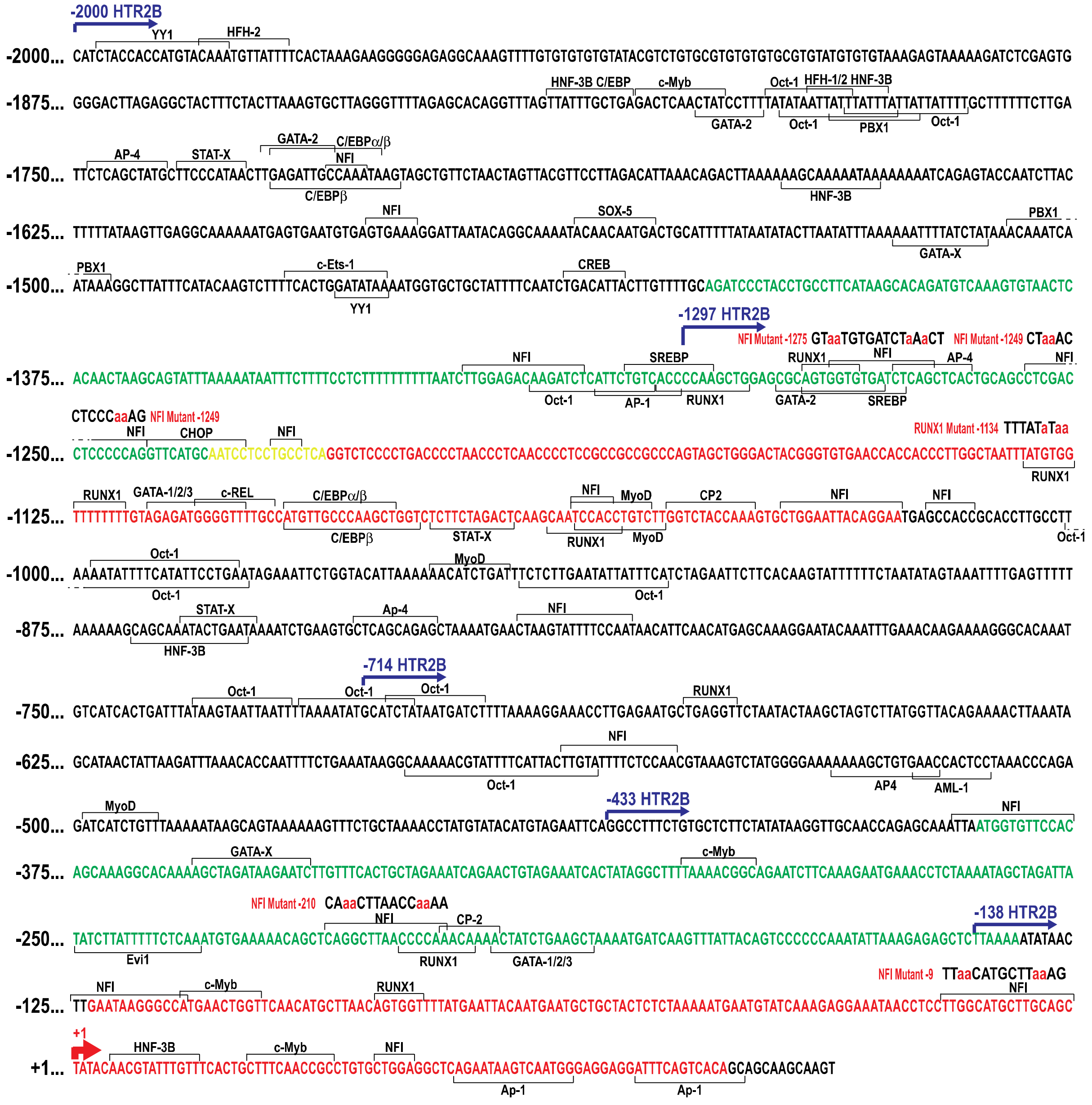

Supplementary Figure S1. DNA sequence of the human HTR2B promoter and 5' flanking region

DNA sequence of the human *HTR2B* gene promoter that depicts the position of the predicted mRNA start site (red arrow). Curved blue arrows with numbers above them (-2000HTR2B, -1297HTR2B, etc…) denote the 5' position of the *HTR2B* promoter segment cloned into pCATbasic and used for transfection. Sequence positions indicated on the left are relative to the predicted mRNA start site. Brackets indicate the position of each putative transcription factor target site identified by the TFSEARCH program. The areas covered by the primers used for ChIP-Seq analyses are indicated in red and green. The mutated oligonucleotides used for site directed mutagenesis of the NFI sites are also shown.

Supplementary tables

Supplementary Table S1. DNA sequence of the primers and double-stranded oligonucleotides

Primers used for qPCR analyses

Gene	Forward Primer (5'-3')<br>Reverse Primer (5'-3')	Genebank #
HTR2B	TCTTTTCAACCGCATCCATCA<br>TGCTGTAGCCCGTGAGTTATA	NM_000867
NFIA	CAGGTGTGATCCTCCTTGAG<br>TTAACTGCTGACTGCTGAACC	NM_005595
NFIB	TCCTGCCAAGAATCCTCCAG<br>TTGGTGGAGAAGACAGAGACC	NM_ 001282787
NFIC	CGCACACACTCAGGAGGAA<br>AGGCGGAGAGGAGATGAATAA	NM_205843
NFIX	TCTGGAATGTGACGGAGCTG<br>CTGTCATCGATGGACTTGGG	NM_002501
RUNX1	GCAACGGGAAATGTGGTCCT<br>GGAGAGAGGGTTCTGGGAT	NM_001754
GAPDH	AAGGTCGGAGTCAACGGAT<br>GGAAGATGGTGATGGGATTTC	NM_002046

Oligonucleotides used as labeled probes or competitors in the EMSAs

Oligonucleotide	Top strand (5'-3')<br>Bottom strand (5'-3')
**AP-1**	GATCCCCGCGTTGAGTCATTCGCCTC<br>GATCGAGGCGAATGACTCAACGCGGG
**NFI**	TTATTTTGGATTGAAGCCAATATGAG<br>CTCATATTGGCTTCAATCCAAAATAA
**RUNX1**	GATCGGCTAATTTATGTGGTTTTTTTGTAGA<br>GATCTCTACAAAAAAACCACATAAATTAGCC
**RUNX1 Mutant**	GATCGGCTAATTTATTTTTTTTTTTTGTAGA<br>GATCTCTACAAAAAAAAAAAAATAAATTAGCC
**-9 HTR2B NF1**	GATCAACCTCCTTGGCATGCTTGCAGCTATACAA<br>GATCTTGTATAGCTGCAAGCATGCCAAGGAGGTT
**-9 HTR2B NF1 Mutant**	GATCAACCTCCTTAACATGCTTAAAGCTATACAA<br>GATCTTGTATAGCTTTAAGCATGTTAAGGAGGTT
**-210 HTR2B NF1**	GATCAACAGCTCAGGCTTAACCCCAAACAAAACT<br>GATCAGTTTTGTTTGGGGTTAAGCCTGAGCTGTT
**-210 HTR2B NF1 Mutant**	GATCAACAGCTCAAACTTAACCAAAAACAAAACT<br>GATCAGTTTTGTTTTTGGTTAAGTTTGAGCTGTT

Oligonucleotides used for site-directed mutagenesis

Mutated HTR2B site	Top strand (5'-3')<br>Bottom strand (5'-3')
**-9 NFI**	CAAAGAGGAAATAACCTCCTTAACATGCTTAAAGCTATACAACGTATTTGTTTC<br>GAAACAAATACGTTGTATAGCTTTAAGCATGTTAAGGAGGTTATTTCCTCTTTG
**-210 NFI**	CTCAAATGTGAAAAACAGCTCAAACTTAACCAAAAACAAAACTATCTGAAGCT<br>AGCTTCAGATAGTTTTGTTTTTGGTTAAGTTTGAGCTGTTTTTCACATTTGAG
**-1249 NFI**	CTCACTGCAGCCTAAACCTCCCAAAGGTTCATGCAATCCTCC<br>GGAGGATTGCATGAACCTTTGGGAGGTTTAGGCTGCAGTGAG
**-1275 NFI**	CAAGCTGGAGCGCAGTAATGTGATCTAAACTCACTGCAGCCT<br>AGGCTGCAGTGAGTTTAGATCACATTACTGCGCTCCAGCTTG
**-1134 RUNX1**	CTGACCCCTAGGCTAATTTATTTTTTTTTTTTTGTAGAGATGGGGTTTTG<br>CAAAACCCCATCTCTACAAAAAAAAAAAAATAAATTAGCCTAGGGGTCAG

Oligonucleotides used for ChIP-qPCR

Oligonucleotide	Top strand (5'-3')<br>Bottom strand (5'-3')
**NFI: -1420/-1229**	AGATCCCTACCTGCCTTCATA<br>GAGGCAGGAGGATTGCATGA
**NFI: -387/-133**	GCAGAATCTTCAAAGAA GAAACC<br>ACCAGTTCATGGCCCTTATTC
**NFI: -123/+83**	GAATAAGGGCCATGAACTGGT<br>TGCTGTGACTGAAATCCTCCT
**RUNX1: -1234/-1022**	AATCCTCCTGCCTCAGGTC<br>CCTGTAATTCCAGCACTTTGGT
**NFI+RUNX1: -1419/-1052**	AGATCCCTACCTGCCTTCATA<br>AGACAGGTGGATTGCTTGAGT

Supplementary references

1. Duval, C.; Zaniolo, K.; Leclerc, S.; Salesse, C.; Guerin, S. L., Characterization of the human alpha9 integrin subunit gene: Promoter analysis and transcriptional regulation in ocular cells. *Experimental eye research* **2015,** 135, 146-63.
2. Ouellet, S.; Vigneault, F.; Lessard, M.; Leclerc, S.; Drouin, R.; Guerin, S. L., Transcriptional regulation of the cyclin-dependent kinase inhibitor 1A (p21) gene by NFI in proliferating human cells. *Nucleic Acids Res* **2006,** 34, (22), 6472-87.
3. Gaudreault, M.; Vigneault, F.; Leclerc, S.; Guerin, S. L., Laminin reduces expression of the human alpha6 integrin subunit gene by altering the level of the transcription factors Sp1 and Sp3. *Invest Ophthalmol Vis Sci* **2007,** 48, (8), 3490-505.
